# Dual-Task Gait as a Predictive Tool for Cognitive Impairment in Older Adults: A Systematic Review

**DOI:** 10.3389/fnagi.2021.769462

**Published:** 2021-12-24

**Authors:** Felipe Ramírez, Myriam Gutiérrez

**Affiliations:** ^1^Programa Magíster en Kinesiología Gerontológica, Facultad de Ciencias, Universidad Mayor, Santiago, Chile; ^2^Escuela de Kinesiología, Facultad de Ciencias de la Salud, Universidad de Las Américas, Santiago, Chile; ^3^Centro de Estudio del Movimiento Humano, Escuela de Kinesiología, Facultad de Odontología y Salud, Universidad Diego Portales, Santiago, Chile; ^4^Unidad de Cerebro Saludable, Hospital Clínico Universidad de Chile, Santiago, Chile

**Keywords:** cognitive-motor task, cognitive decline, screening tool, aging, dementia

## Abstract

The use of the dual-task model as a cognitive-motor interface has been extensively investigated in cross-sectional studies as a training task in cognitive impairment. However, few existing longitudinal studies prove the usefulness of this tool as a clinical marker of cognitive impairment in older people. What is the evidence in prospective studies about dual-task gait as a predictor of cognitive impairment in older adults? This study aims to review and discuss the current state of knowledge in prospective studies on the use of dual-task gait as a predictive tool for cognitive impairment in older adults. The methodology used was a systematic review, according to the PRISMA criteria for the search, summarize and report. A search in 3 databases (Pubmed, Web of Science, and Scopus) was carried out until April 2021. The search terms used were: “(gait OR walking) AND (cognitive decline) AND (dual-task) AND (follow-up OR longitudinal OR long-term OR prospective OR cohort OR predict).” We included prospective research articles with older people with cognitive evaluation at the beginning and the end of the follow-up and dual-task gait paradigm as initial evaluation associated with the presentation of cognitive impairment prediction using any dual-task gait parameters. After exclusion criteria, 12 studies were reviewed. The results indicate that eight studies consider dual-task gait parameters a useful cognitive-motor tool, finding that some of the evaluated parameters of dual-task gait significantly correlate with cognitive impairment over time. The most promising DT parameters associated with cognitive impairment prediction seem to be gait speed, speed cost, DT time, numbers of words during DT, among others. In sum, this study reviews the variety of dual-task gait parameters and their relevance as a simple tool for early cognitive impairment screening, opening a diagnostic window for the screening of cognitive impairment in older people.

## Introduction

World population aging brings up several challenges, including an increase in cognitive impairment and dementia cases. There are 10 million new cases of dementia every year worldwide (WHO, 2020), and this number will double in 20 years (Ferri et al., 2005). The prevalence of dementia is rapidly increasing in middle and low-income countries (Prince et al., 2013). In Chile, 1% of the population has dementia, and it is projected to triple by 2050 (Ministerio de Salud, 2010, 2017). Cognitive impairment and dementia are a priority in public health planning programs considering their high economic and social cost (Alzheimer's Research UK, 2017). It is associated with a high burden on formal and informal caregivers (Brodaty et al., 2013). In Chile, this economic and social cost is carried mainly by female relatives, diminishing their job opportunities (Hojman et al., 2017). Considering the progression from cognitive impairment to dementia, it is crucial to identify risk factors for cognitive impairment for early prevention, proper management, and optimizing the quality of life of the patient and family (Organización Panamericana de la Salud, 2020). Early stages include mild cognitive impairment (MCI), with a five-fold higher risk of developing dementia (Petersen, 2011). MCI can be diagnosed by neuropsychological tests such as the Mini-Mental State Examination (MMSE) (Folstein et al., 1975) or the Montreal Cognitive Assessment (MoCA) (Nasreddine et al., 2005). However, its evolution is difficult to predict because of the variety in the clinical manifestations and the speed of the cognitive functions change and decline progression (Storandt et al., 2002). For this reason, screening tools for early stages of cognitive impairment assessment are critical.

Cognitive impairment diagnosis is fundamentally clinical. Complementary tests to predict the risk among life courses are also relevant (Livingston et al., 2020). They include medical history evaluation, blood tests, neuropsychological tests, brain imaging, cerebrospinal fluid analysis, positron emission tomography (PET), and biomarkers as indexes for health (US National Library of Medicine, 2016). For diagnosis and progression, neuropathological biomarkers include amyloid β1-42 (inversely related to amyloid burden in the brain), total tau (for neuronal degeneration), and phospho-tau (for the density of neurofibrillary tangles) (Bayer, 2018). Nevertheless, these evaluations are expensive, time-consuming, invasive, and not always available, making it even more complex to establish a diagnosis and prognosis of cognitive impairment (Tolonen et al., 2018). Therefore, alternative clinical tools are required to help to identify early stages of cognitive impairment (Laske et al., 2014).

In the search for new screening tools for cognitive impairment, non-cognitive markers have gained relevance (Montero-Odasso et al., 2020). One is the simultaneously cognitive and motor performance, named dual-task (DT). DT is the simultaneous performance of these two tasks. The most common DT paradigm is the use of a cognitive task that involves executive functions, plus a motor task that challenges the performance of the first task (Petrigna et al., 2019), such as gait. This assessment considers shared neural networks between movement and cognition (Waite et al., 2005; Aggarwal et al., 2006; Liu-Ambrose et al., 2008; Verghese et al., 2008; Buchman and Bennett, 2011; Mielke et al., 2013; Beauchet et al., 2016). Indeed, cerebral image-based evidence shows functional and structural correlates between motor control and cognition, such as prefrontal and temporal brain regions activated during motion (Scherder et al., 2007; Montero-Odasso et al., 2012b; Rosano et al., 2012; Rosso et al., 2013). Also, cognitive functions such as attention, memory, and executive function are necessary during walking (Camicioli et al., 1997; Kluger et al., 1997; Sheridan et al., 2003; Al-Yahya et al., 2011). Furthermore, hippocampal activity is critical during spatial memory, and it is necessary for visual, somatosensory, and vestibular integration during spatial orientation in gait (Scherder et al., 2007). Moreover, it has been observed that MCI and decreased gait speed share a decrease in hippocampal volume (Rosso et al., 2017).

In older people, neuroimaging studies have shown greater use of frontal-subcortical circuits during gait (Malouin et al., 2003; Al-Yahya et al., 2011). When using a dual-task gait paradigm, older people show higher activation of this area than younger people (Ohsugi et al., 2013). Evidence of the use of motor parameters for the MCI screening tool has positioned gait speed as a good marker (Quan et al., 2017). Particularly, MCI accompanied by slow gait speed is a more significant risk factor for dementia than slow gait speed or cognitive impairment alone (Doi et al., 2018). This evidence supports the increased diagnostic value of dual-task assessment over the evaluation of motor and cognitive capacities alone.

There are several models of dual-task for assessment and treatment. Gait as a motor task using usual pace gait (UPG) in straight-line or the Timed Up and Go test (TUG), plus naming animals or counting backward as the cognitive task, are examples of dual-task gait (Leone et al., 2020). This ability to stress the cognitive and motor system makes the dual-task an ideal tool to reflect the motor-cognitive interface (Camicioli et al., 1997; Al-Yahya et al., 2011; Albers et al., 2016; Macaulay et al., 2017; Smith et al., 2017).

Furthermore, considering early changes in functional performance activities of daily living (ADL) in MCI (Burton et al., 2009), it has even been observed that gait parameters can also be observed before cognitive changes (Mielke et al., 2013; Best et al., 2016). Conventional functional assessment tests would not be sensitive to detect these changes (Gillain et al., 2009). On the other hand, assessing gait alterations can predict cognitive functions (Scherder et al., 2007). This idea is supported by the presence of gait disorders associated with hippocampal degeneration and the nigrostriatal system (Scherder et al., 2007) and due to the motor impairment that accompanies cognitive impairment before the development of dementia (Montero-odasso et al., 2018). Indeed, a brain with less functional reserve is more exposed to overload due to the activation of additional areas during gait (Scherder et al., 2007; Montero-Odasso et al., 2012b; Montero-odasso et al., 2014), especially by making this task more complex with an additional task (Al-Yahya et al., 2011; Smith et al., 2017).

Previous studies have used gait speed (GS) as the main motor parameter of dual-task gait. These studies consider the significant relationship between increased stride variability and cognitive impairment (Laske et al., 2014). Other DT parameters associated with cognitive impairment prediction include instrumental biomechanical variables such as step symmetry, speed cost, swing time, stride time, among others.

Increasing evidence supports the use of dual-task gait as a tool to discriminate the progression between different levels of cognitive impairment (Laske et al., 2014; Montero-odasso et al., 2014; Macaulay et al., 2017; Åhman et al., 2020b; Latorre et al., 2020). It has been proposed as a predictive assessment of the progression from MCI to dementia (Laske et al., 2014; Bahureksa et al., 2017; Chiaramonte and Cioni, 2021). The decrease in gait speed while dual-task gait is associated with deficits in executive, attention, and memory processes, as well as the progression from a healthy state or MCI to dementia (Camicioli et al., 1997; Bootsma-van der Wiel et al., 2003; Hausdorff et al., 2005; Montero-odasso et al., 2009; Chiaramonte and Cioni, 2021).

It has also been reported that gait speed in the dual-task gait paradigm could be more valuable than gait speed in a simple task (only gait) to discriminate between healthy people and those with MCI (Macaulay et al., 2017), explained by an activation overlap of brain areas that interfere with the execution of the double task (Bürki et al., 2017). Furthermore, dual-task is most useful in older people since walking alone can be complex (Hausdorff et al., 2005).

All these antecedents taken together make dual-task gait a promising tool for diagnosing cognitive impairment. However, few longitudinal studies test a dual-task gait paradigm to predict cognitive impairment and dementia in older people. What is the evidence in prospective studies about dual-task gait as a predictor of cognitive impairment in older adults? This study aims to review and discuss the current stage of knowledge on prospective studies of the dual-task gait model as a predictor of cognitive impairment in older people.

## Methods

The strategy for developing this systematic review followed the Preferred Reporting Items for Systematic Reviews and Meta-Analysis (PRISMA) protocol (Moher et al., 2009) for the search, summarize, and report (Page et al., 2021). We defined the research question regarding populations, interventions, comparators, outcomes, and study designs (PICOS) as follows: What is the evidence in prospective studies about dual-task gait as a predictor of cognitive impairment in older adults? Is dual-task gait able to predict cognitive impairment in older adults? For addressing this question, during March 14, 2021, a pilot search strategy was carried out in the Scopus, Web of Science, and PubMed databases using the following search terms: (gait) AND (cognitive dysfunction) AND (dual-task) AND (follow-up OR longitudinal OR long-term OR prospective OR cohort OR predict) AND (2012–2021). It showed 291 possible candidate studies.

**Figure 1:**
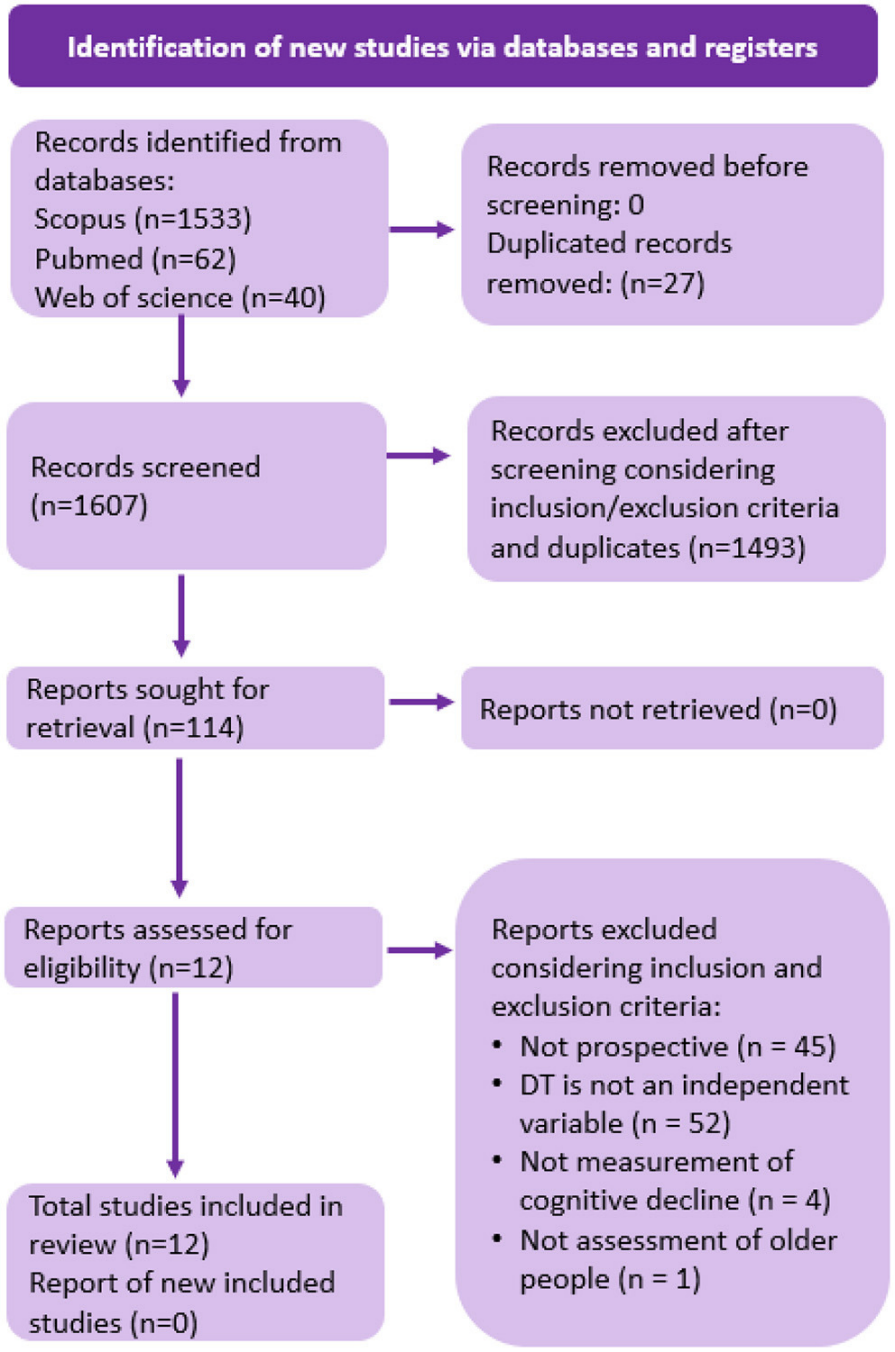
Article selection process. This figure compiles the selected studies according to PRISMA 2020 flow diagram for systematic reviews. The boxes provide the selection process according to search methods (see text). There were 1,635 initial records identified from databases with 27 duplicates. After title screening based on inclusion/exclusion criteria and duplicates, 114 reports were sought for retrieval. After assessing eligibility, 12 studies were included in this review (Page et al., 2021).

For a more comprehensive review, we eliminated time restrictions. We performed a final search during April 20, 2021, considering medical topic headings (MeSH) and search words detailed in Table 1: “(gait OR walking) AND (cognitive decline) AND (dual-task) AND (follow-up OR longitudinal OR long-term OR prospective OR cohort OR predict).” It showed 1,607 records screened. We included research articles written in English and Spanish with a prospective design which sample were older people (60 years and over as stated by World Health Organization) able to perform the required test independently. For a comprehensive analysis of outcomes, we considered the use of cognitive evaluation at the beginning and the end of the follow-up (dependent variable) and dual-task gait paradigm as initial evaluation (independent variable) associated with the presentation of cognitive impairment prediction using any dual-task gait parameters. Cognitive decline was defined as worsening performance in the neuropsychological assessment over time. Cognitive impairment was considered a stage (from mild to moderate) between normal aging cognitive decline and a pathologic cognitive decline. It excluded severe cognitive impairment status or major neurocognitive disorder (dementia) at baseline since this study aims to predict cognitive impairment progression. We excluded studies with subjects with chronic pathologies that markedly affect mobility because it would limit them from performing the required motor test based on literature (e.g., Parkinson's disease, Huntington's disease, multiple sclerosis, cerebrovascular accident). We also excluded case studies and research in an animal model. This research included only original articles with a prospective design for older people to address the research question. After applying inclusion and exclusion criteria and manually removing duplicated articles, 12 articles were selected for this review (Figure 1). To improve the completeness of reporting, organization of results, synthesis of the findings, and review's utility, we consulted the PRISMA reporting checklist and abstract checklist (Page et al., 2021).

**Table 1:** Search terms according to PICOS.

**Population**	**Intervention or exposure**	**Comparison**	**Outcome**	**Study**
Aged (MeSH)	Dual-task	(none)	Cognitive impairment	Only articles
Older adults (MeSH)	Dual-task		Cognitive impairment	Longitudinal
Older people (MeSH)	Gait (MeSH)		Cognitive dysfunction	Follow-up
Elderly population (MeSH)	Gait (MeSH)		Predict	Long-term
Cognitive impairment (MeSH)	Walking (MeSH)			Prospective
Dementia (MeSH)	Walking (MeSH)			Cohort
				NOT intervention

*This table compiles the used search terms for articles selection according to Populations, Interventions, Comparators, Outcomes, and Study designs (PICOS). MeSH, medical topic headings*.

### Quality Assessment

Two researchers performed the discrimination and selection of studies. They independently reviewed abstracts and registered findings into a database. Then hand-searched and selected full relevant articles and documents for data extraction using the abovementioned preset criteria. There was agreement in the selected studies. Discrepancies were solved through discussion with a third reviewer. The contributions of both authors are described in the author's contributions section.

**Figure 2:**
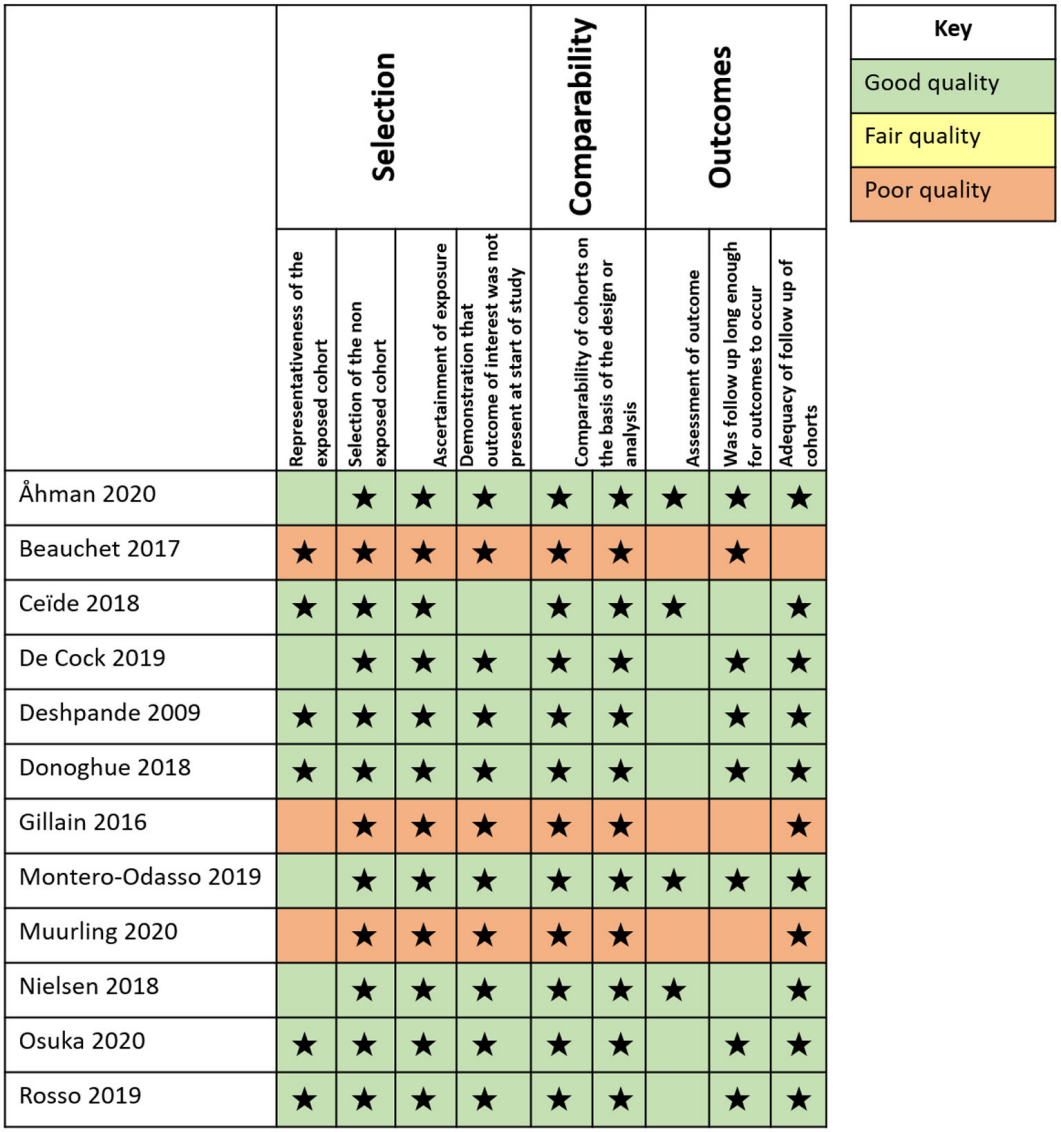
Bias assessment. Diagram presenting the risk of bias assessment of the studies included according to the Newcastle-Ottawa quality assessment scale (NOS). The number of stars indicates the quality of the reviewed article: Good quality: 3 or 4 stars in selection domain AND 1 or 2 stars in comparability domain AND 2 or 3 stars in outcome/exposure domain; Fair quality: 2 stars in selection domain AND 1 or 2 stars in comparability domain AND 2 or 3 stars in outcome/exposure domain; Poor quality: 0 or 1 star in selection domain OR 0 stars in comparability domain OR 0 or 1 stars in outcome/exposure domain (Wells et al., 2000).

To assess the risk of bias assessment of the studies included, we included Figure 2 based on Newcastle-Ottawa quality assessment scale for assessing the risk of bias of longitudinal studies (Wells et al., 2000).

## Results

This review includes 12 studies (Table 2). Characteristics include a follow-up time from a minimum of 1 year and a maximum of 9 years (average 3.54 years). The sample size included a minimum of 13 (pilot study) and a maximum of 2,250 subjects. The participants were older people aged 60 years and older (average age 71.4) who did not require assistance to perform motor or cognitive tests. The initial cognitive state of the participants included: 25.0% (3 studies) only “with MCI” (Gillain et al., 2016; Montero-Odasso et al., 2017; Åhman et al., 2020a); 25.0% (3 studies) “without dementia” (Beauchet et al., 2017; Ceïde et al., 2018; Donoghue et al., 2018); 16.7% (2 studies) “without cognitive impairment” (Rosso et al., 2019; Osuka et al., 2020a); and 33.3% (4 studies) “cognitively healthy” (CH) people and with “some degree of cognitive impairment” (Deshpande et al., 2009; Nielsen et al., 2018; De Cock et al., 2019; Muurling et al., 2020).

**Table 2:** Main results of reviewed articles.

**Article**	**Main results**
Åhman et al. (2020a)	The following variables showed significant association with incidence of dementia in the general sample: - Naming animals DT time (s) - Numbers of words recited in naming animals DT (*n*) - Words recited per 10 s during the naming animals DT (*n*) - Counting backward DT time (s) - Number of words recited in the reciting months backward DT (*n*) - Words recited per 10 s during the reciting months backward DT (*n*) The following variables were strongly associated with the incidence of dementia in younger than 72 years subsample: - Words recited per 10 s during the naming animals DT (*n*)
Beauchet et al. (2017)	Significative association in increased delta MMSE with: - Mean value of stride time in naming animals DT - CoV of stride time in naming animals DT - Delta CoV in naming animas DT
Ceïde et al. (2018)	Significative association with the incident and vascular dementia: - Swing time SD in DT
De Cock et al. (2019)	In the general sample, the initial MCI group: - Step width (cm) in “UPG + counting down from 50 in steps of two” DT differentiated between eventual AD+FTD (Alzheimer's disease and frontotemporal dementia) and non-demented people. - Gait speed (cm/s), normalized gait speed (m/s) and normalized steps/meter (steps/m) in “UPG + naming animals” DT differentiated between at risk for VascD+LBD (vascular dementia and Lewy Body dementia) and non-demented people. In older than 70 years subsample, the initial MCI group: - Step width (cm) in “UPG + counting down from 50 in steps of two” DT differentiated between non-demented and eventual AD + FTD. - Gait speed (cm/s), normalized gait speed (m/s) and normalized steps/meter (steps/m) differentiated between VascD + LBD and non-demented people.
Deshpande et al. (2009)	There was no significant association between DT variables and cognitive decline.
Donoghue et al. (2018)	There was no significant association between DT variables and cognitive decline.
Gillain et al. (2016)	Significative differences between demented MCI group and non-demented group in: - Gait speed (m/s) - Symmetry (absolute value)
Montero-Odasso et al. (2017)	Significative association between dementia progression and: - Counting backward from 100 DT gait speed (cm/s, continuous variable) - Naming animals DT gait speed (cm/s, continuous variable) - Naming animals DT gait speed cost (%, continuous variable)
Muurling et al. (2020)	There was no significant association between DT variables and cognitive decline
Nielsen et al. (2018)	There was no significant association between DT variables and cognitive decline
Osuka et al. (2020a)	Significative association between cognitive decline and the highest tertile of S-TMT time (s)
Rosso et al. (2019)	Significative association between cognitive decline progression and: - DT gait speed percent change (%) - DT gait speed (0.1 m/s)

*This table summarizes the results of the 12 analyzed prospective studies regarding dual-task prediction. DT, dual-task; TUG, Timed up and Go test; UPG, Usual pace gait; CoV, Coefficient of variation; S-TMT, Stepping Trail Making Test (consisting of a test where the participant is asked to walk inside a 1 × 1 m square divided into 16 squares of the same size numbered from 1 to 16 in an established order, stepping the squares in consecutive order) (Osuka et al., 2020b). MMSE, MiniMental State Scale; MCI, Mild Cognitive Impairment; SCI, Subjective cognitive impairment; AD, Alzheimer's disease. DT cost was calculated from the formula: [(usual pace gait parameter – gait parameter)/usual pace gait parameter] × 100. CoV = [(standard deviation/mean) × 100]. Delta mean value, and delta CoV were calculated from the formula: [dual-task–single task/(dual-task + single task)/2] × 100. Delta MMSE was calculated from the formula: [baseline MMSE–MMSE at 5 years of follow-up/(baseline MMSE + MMSE at 5 years of follow-up)/2] × 100*.

According to 66.7% of the reviewed articles (8 studies), the dual-task (DT) paradigm has predictive value for cognitive impairment (Gillain et al., 2016; Beauchet et al., 2017; Montero-Odasso et al., 2017; Ceïde et al., 2018; De Cock et al., 2019; Rosso et al., 2019; Åhman et al., 2020a; Osuka et al., 2020a). Contrariwise, 30.7% (4 studies) did not find a significant relationship between the DT variables evaluated and the appearance of cognitive impairment (Deshpande et al., 2009; Donoghue et al., 2018; Nielsen et al., 2018; Muurling et al., 2020).

The DT parameters measured in the studies that analyzed gait as a motor task were: gait speed, gait speed cost, cadence, cadence cost, steps/meter, steps/meter cost, step, variability of step length, step width, step width cost, variability of step width, variability of step width cost, swing percentage, stance percentage, stance time, stance time variability, swing time, swing time variability, swing time variability cost, cycle time variability, cycle time variability cost, step time, stride time, stride time variability, the mean value in usual gait stride time, coefficient of variability (CoV) in usual gait stride time, stride time mean value, delta stride time mean value, delta stride time CoV, regularity, and symmetry between steps and gait speed (GS). The main results in each analyzed study, including a significative association between DT gait parameters, are summarized in Table 2.

GS was the most used variable in DT models that used the usual gait as a motor task. Beauchet et al. (2017) used stride time only. However, GS was not always associated with the appearance of cognitive impairment (Deshpande et al., 2009; Donoghue et al., 2018; Muurling et al., 2020). Significant associations with cognitive impairment over time were found considering other DT motor variables, such as coefficient of variability (CoV) stride time, delta CoV stride time, variability, swing time, stride width, steps per meter, time variability of the gait cycle, step width cost, step symmetry, and speed cost as relevant gait variables.

Considering unconventional motor tasks, Muurling et al. (2020) used a gait test consisting of getting up from a chair, walking 5 meters as quickly and comfortably as possible, turning a cone, turning the chair, back to the cone, and sitting, forming an 8. This test is similar to the TUG and simple gait tests. In contrast, Osuka et al. (2020a) used a motor test created by their team, consisting of a 1 × 1 m^2^ with 16 squares inside, numbered from 1 to 16, where the evaluated subject is asked to step on the numbers consecutively (Osuka et al., 2020b). Comparing both tests, Murling measured gait parameters, while Osuka evaluated task execution time.

The cognitive tasks of the DT paradigm considered primarily three capabilities: working memory (reciting the months of the year backward, subtraction, reciting the alphabet by alternating letters, counting backward, and numbers counted forward during DT gait), verbal fluency function (naming animals, reciting names, and the number of words during DT gait), attention and visuospatial ability (Trail making test and visuospatial task). It is worth mentioning that Åhman et al. (2020b), who used a verbal fluency test as a cognitive task, measured the number of months named during performance time × 10 and the number of animals named during performance time × 10. This test achieved a strong association with cognitive impairment for the total population (OR = 4.06, 95% CI 2.28–7.23, *p* < 0.001) and for people under 72 years of age (OR = 20.9, 95% CI 3.29 −133.13, p = 0.001). This result was not obtained with other variables. Also, it is worth mentioning that the study by Nielsen et al. (2018) that only used time (OR = 0.222, 95% CI 0.045–1.094) and cost (OR = 0.682, 95% CI 0.122–3.825) found no significant relationship between its variables and cognitive impairment over time.

TUG was used as the motor task in 2 studies (Nielsen et al., 2018; Åhman et al., 2020a). The motor task parameters considered included: DT time (s), GS (cm / s), DT cost (%), and qualitative performance of DT. The cognitive parameters that were associated with cognitive impairment are: naming animals DT time (s), numbers of words recited in naming animals DT (*n*), words recited per 10 s during the naming animals DT (*n*), counting backward DT time (s), number of words recited in the reciting months backward DT (*n*), words recited per 10 s during the reciting months backward DT (*n*).

The DT parameters used (Table 2) as predictors for cognitive impairment, the protocols employed, the populations included, and how the outcome was defined are summarized in Table 3.

**Table 3:** Summary of reviewed articles.

**Article**	**Participants** ***N*** mean age^*^ **% female** **Initial cognitive state**	**Time of the follow-up**	**DT paradigm** **(motor task + cognitive task)**	**Cognitive assessment at baseline and follow-up**	**DT parameters**
Åhman et al. (2020a)	17271.0 years 45.3% MCI or SCI people from a memory clinic	2.5 years	TUG + naming animals TUG + reciting months backward	MMSE, 7 Min Screen neurocognitive test (specifically Clock Drawing and Verbal Fluency tests).	• Simple TUG time (s) • Naming animals DT time (s) • Naming animals DT time cost (%) • Numbers of words recited in naming animals DT (n) • Words recited per 10 s during the naming animals DT (n) • Counting backward DT time (s) • Counting backward DT time cost (%) • Number of words recited in the reciting months backward DT (n) • Words recited per 10 s during the reciting months backward DT (n)
Beauchet et al. (2017)	56 68.9 years 46.4% Community-dwelling older people without dementia	5 years (4.8 ± 0.7 years)	UGS + naming animals UGS + counting backward since 50	MMSE.	• Initial stride time characteristics: • Mean value and CoV in usual gait • Mean value, CoV; delta mean value, and delta CoV in counting backward DT • Mean value, CoV; delta mean value, and delta CoV in verbal fluency DT
Ceïde et al. (2018)	1,156 78.3 years 60.7% Community-dwelling older adults without dementia	1.9 mean years	UPG + reciting alternate letters of the alphabet (instructed paying attention equally in both tasks)	Short form of Wechsler Adult Intelligence Scale-Revised (WAIS-R), Mill Hill Vocabulary scale, FCSRT, BIMC.	• DT parameters: • Speed (cm/s) • Cadence (step/min) • Step length (cm) • Swing percentage (%) • Stance percentage (%) • Swing time SD • Step time SD • DT gait domains: • Rhythm • Variability • Pace
De Cock et al. (2019)	433 80.0 years 45.7% Older people from a memory clinic with healthy cognition, MCI, and incident dementia	5 years	UPG + naming animals UPG + counting down from 50 in steps of two	MMSE, ACE, NPI-Q, CDR.	• From usual pace gait, fast-paced gait, slow-paced gait, naming animals DT gait, counting backward in steps of two DT gait were obtained the following variables: • In pace gait domain: • Gait speed (cm/s) • Normalized gait speed^***^ (m/s) • Cadence (steps/min) • Steps/meters (steps/m) • Step length (m) • Normalized steps per meter^***^ (steps/m) • Postural control gait domain: • Step width (cm) • Step width variability (%) • Variability gait domain: • Swing time variability (%) • Cycle time variability (%) • Additionally, from both DT paradigms were obtained: • DT gait speed cost (%) • DT cadence cost (%) • DT step width cost (%) • DT width step variability cost (%) • DT cycle time variability cost (%) • DT swing time variability cost (%) • DT steps/meter cost (%) • Normalized DT steps/meter cost^***^ (%) • Counted numbers during 10 meters in counting backward DT (discrete number) • Counted animals during 10 meters in naming animals DT (discrete number)
Deshpande et al. (2009)	660 74.6 (5.3) 54.2% Community-dwelling older people	3 years	UPG + naming animals	MMSE	• Gait speed (m/s) of: • UPG • Fast-paced gait • DT gait
Donoghue et al. (2018)	2,250 72.4 years^**^ 52.0%^**^ Community-dwelling older people without dementia	5.9 years	UPG + reciting alternate letters of the alphabet	MMSE, Verbal Fluency, Immediate recall, Delayed recall, MoCA, Color trails 1 time, Color trials 2 times, Color trials time difference, Cognitive response time, Movement time, Total response time, SART mean response time (ms), SART SD (ms), SART coefficient of variation (%), SART errors of commission (n), SART errors of omission (*n*)	• TUG time (s) • UPG speed (cm/s) • DT speed (cm/s)
Gillain et al. (2016)	13 73.1 years^**^ 46.2%^**^ MCI older people from a memory clinic	4 years	UPG + counting backward from 50	MMSE, CDR.	• Variables obtained from UPG and DT: • Gait speed (m/s) • Regularity (absolute value) • Symmetry (absolute value)
Montero-Odasso et al. (2017)	112 76.0 years 49.1% Community-dwelling older people with MCI	2 years (12–76 months)	UPG + naming animals UPG + counting backward from 100 UPG + subtracting several sevens from 100	MMSE, CDR, TMT-A, TMT-B, Rey auditory verbal Learning test, Boston naming test, Digit Span Forward, Digit Span backward, Letter-Number Sequencing test.	Single task gait speed (cm/s, continuous variable) • Counting backward from 100 DT gait speed (cm/s, continuous variable) • Subtracting several sevens from 100 DT gait speed (cm/s, continuous variable) • Naming animals DT gait speed (cm/s, continuous variable) • Counting backward from 100 DT gait speed cost (%, continuous variable) • Subtracting several sevens from 100 DT gait speed cost (%, continuous variable) • Naming animals DT gait speed cost (%, continuous variable) • Lower gait speed in the single-task (<0.8 m/s, dichotomic variable) • High cost in Counting backward from 100 DT gait speed (>20%, dichotomic variable) • High cost in subtracting several sevens from 100 DT gait speed (>20%, dichotomic variable) • High cost in Naming animals DT gait speed (>20%, dichotomic variable)
Muurling et al. (2020)	142 67.0 years 47% Older people from a memory clinic grouped in healthy cognitively, MCI, and mild dementia.	1.2 years^**^ (1–2 years)	WT8 + counting backward from 100	MMSE, CDR.	• Variables of UPG and DT: • Mean stance time (s) • Mean stride time (s) • Mean swing time (s) • Cadence (steps/min) • Stance time variability (s) • Stride time variability (s) • Swing time variability (s) • Mean step length (m) • Speed (m/s) • Step length variability (m)
Nielsen et al. (2018)	86 72.0 years 20.7% Older people from a memory clinic grouped in cognitively healthy, MCI, and mild dementia	2.5 years^**^ (12–36 months)	TUG + counting backward from 100	MMSE, ACE, CDR.	• TUG time (s) • TUG DT time (s) • TUG DT cost (%) • DT performance (normal, moderate deviation, and severe deviation)
Osuka et al. (2020a)	626 76.0 years 61.8% Community-dwelling older people without cognitive impairment	2 years	S-TMT	MMSE, TMT A.	• S-TMT time divided in tertials: highest, middle and lowest (s) • TMT-A time (s) • UPG speed (m/s)
Rosso et al. (2019)	223 78.7 years 52.5% Community-dwelling older people without cognitive impairment	9 years	UPG + visuospatial clock task	3MS.	• UPG speed (0.1 m/s) • Fast-paced gait speed (0.1 m/s) • Narrow path gait speed (0.1 m/s) • DT gait speed (0.1 m/s) • Fast-paced gait speed cost (%) • Narrow path gait speed cost (%) • DT gait speed cost (%)

*This table summarizes the characteristics of the 12 reviewed prospective studies. Each row is representative of a study. The first column indicates the participant's characteristics (number, sex, age, and cognitive status); the 2nd column indicates the follow-up time in years; the 3rd column summarizes the used DT tasks (motor + cognitive); 4th column summarizes the cognitive assessment performed in each study, and the last column summarizes the main dual-task gait variables that are used as a predictor of cognitive impairment in older adults. DT, dual-task; DT performance: Categories are 3: “Normal” (no notable changes in either gait velocity or performance of the cognitive task during gait); “Moderate deviation” (dual-tasking imply changes in either gait velocity or performance or performance of the cognitive task dual-task); “Severe deviation” (dual-tasking imply either detention in gait when engaging the cognitive task or incapability of performing, according to Nielsen et al., 2018). Motor tasks, TUG: Timed up and Go test; UPG, Usual pace gait; WT8, Walking test (consisting in standing up from a chair, walking 5 m and going around a cone, coming back and going around the chair, the cone again, and sitting down in the chair, forming an 8, at a comfortable, fast pace); S-TMT, Stepping Trail Making Test (consisting of a test where the participant is asked to walk inside a 1 × 1m square divided into 16 squares of the same size numbered from 1 to 16 in an established order, stepping the squares in consecutive order) (Osuka et al., 2020b). Cognitive tests: Visuospatial clock task: It is a test where the participant is asked to indicate if the clock's needles are in the same or the opposite half of the clock, respecting a line between 12 and 6. MMSE, MiniMental State Scale; CDR, Clinical Dementia Rating scale; MoCA, Montreal Cognitive Assessment; SART, Sustained Attention Response Task; TMT, Trail Making Test; FCSRT, Free and Cued Selective Reminding Test; BIMC, Information-Memory-Concentration test; ACE, Addenbrooke's cognitive examination; NPI-Q, Neuropsychiatric Inventory Questionnaire; 3MS, Modified Mini-Mental State; rs, Spearman's rank correlation coefficient; CI, confidence interval; OR, Odd Ratio; HR, Hazard Ratio; MCI, Mild Cognitive impairment; SCI, Subjective cognitive impairment; AD, Alzheimer's disease*.

* *Ages and female percent were rounded to one decimal*

** *This information was calculated indirectly*.

*** *Adjusted by leg length. DT cost was calculated from the formula: [(usual pace gait parameter – gait parameter)/usual pace gait parameter] × 100. CoV: Coefficient of variation was calculated = [(standard deviation/mean) × 100]. Delta mean value, and delta CoV were calculated from the formula: [dual-task – single task/(dual-task + single task)/2] × 100. Delta MMSE was calculated from the formula: [baseline MMSE – MMSE at 5 years of follow-up/(baseline MMSE + MMSE at 5 years of follow-up)/2] × 100*.

## Discussion

The evidence collected in this review suggests that dual-task (DT) gait could be a promising predictor of cognitive impairment since 69.2% of the reviewed articles (8 studies) concluded that a DT paradigm could be helpful as a predictor of cognitive impairment. This finding is consistent with the vast majority of cross-sectional studies that have been done previously regarding the ability of DT to be associated with cognitive impairment (Laske et al., 2014; Montero-odasso et al., 2014; Bahureksa et al., 2017; Macaulay et al., 2017; Åhman et al., 2020b; Latorre et al., 2020). This review's results can help strengthen existing recommendations on the use of DT as an early clinical marker of dementia.

This review highlights gait speed (GS) parameters because it is the most frequently used motor task in the DT paradigm. Most studies consider that GS allows the discrimination between the different states of cognitive impairment (Montero-odasso et al., 2009; Macaulay et al., 2017). Indeed, most studies found a significant association between simple task GS and DT GS with cognitive impairment over time (Gillain et al., 2016; De Cock et al., 2019). This is similar to findings in other studies that associate GS with cognitive performance (Perrochon et al., 2013; Doi et al., 2014; Suk et al., 2020), while other studies found such an association only DT GS (Montero-Odasso et al., 2017; Rosso et al., 2019; Osuka et al., 2020a). However, although a decrease in GS has suggested an elevated risk of cognitive impairment (Quan et al., 2017), the results of this review suggest that simple gait variables such as GS could not be enough to predict cognitive impairment. Three of the analyzed studies that used GS in a DT paradigm did not find a significant association with cognitive impairment progression over time (Deshpande et al., 2009; Ceïde et al., 2018; Muurling et al., 2020). The lack of association can be explained by the sensitivity of the cognitive tasks used in the protocols or simple gait as the motor task, as indicated in Table 2.

These results highlight the higher predictive value of DT gait over simple task gait to discriminate between different stages of cognitive impairment (Montero-odasso et al., 2014; Bahureksa et al., 2017; Macaulay et al., 2017). DT model is consistent with the multidimensional analysis of gait (Aoki et al., 2019; Ehsani et al., 2019) or combining gait variables with cognitive tests, which allows gait to improve the sensitivity of detection of cognitive impairment during DT gait (Speechley et al., 2020).

Additionally, previous research agrees that stride length and step length are good discriminators of the different stages of cognitive impairment (Bahureksa et al., 2017; Latorre et al., 2020). These results are similar to previous studies in stride length in DT in healthy people vs. MCI (Gillain et al., 2009; Maquet et al., 2010). However, three of the reviewed studies did not find a significant association with cognitive impairment over time (Ceïde et al., 2018; De Cock et al., 2019; Muurling et al., 2020).

On the other hand, cadence seems not to be a not good indicator in three studies (Al-Yahya et al., 2011). This can be explained because the association between gait and the subcortical structures affected in the studied populations of the three studies (Ceïde et al., 2018; De Cock et al., 2019; Muurling et al., 2020). The variability of the length of the steps and the stride has been considered a good marker of cognitive impairment (Montero-Odasso et al., 2012a; Tarnanas et al., 2015). It is consistent with Beauchet et al. (2017), who used a DT model of walking and naming animals. There is still no clarity about the length of stride since it has also been seen that it has poor discriminating power for different groups of MCI (Montero-odasso et al., 2014). This DT paradigm is similar to the walking and counting backward tasks used by Muurling et al. (2020) for different states of cognitive impairment (Montero-Odasso et al., 2012a; Tarnanas et al., 2015). According to other authors, using a test that involves walking but also considers getting up, turning, and sitting down could decrease the sensitivity of the assessment (Muurling et al., 2020).

Conversely, DT GS could not be a significant parameter associated with cognitive impairment according to 30.7% of the reviewed studies. Indeed, they did not find a statistically significant association between the initial performance of DT and the change in the cognitive performance of the population evaluated after a specific time (Nascimbeni et al., 2015). In half of these studies (Nielsen et al., 2018; Muurling et al., 2020), a DT paradigm consisted of walking and counting backward one at a time. These tasks combined could be considered a low-cost DT paradigm (Montero-odasso et al., 2014), which implicates a poor, challenging cognitive task, that can be not sensitive enough.

Previous studies have shown the sensitivity of DT to discriminate between different stages of cognitive impairment, such as backward spelling when walking (Macaulay et al., 2017) or holding a glass of water when walking (Suk et al., 2020). But these models have not been included in this review since they used a cross-sectional design. It would be interesting to carry out prospective studies with these DT models to evaluate their predictive value as a marker of cognitive impairment. Indeed, DT has been used to discriminate cognitive decline in patients with dementia (Chiaramonte and Cioni, 2021).

Diverse cognitive components should accompany the use of diverse gait parameters since it has been observed that each gait variable can be a marker of specific cognitive variables (Jayakody et al., 2019). The cognitive task “count backward” has shown a significant relationship with cognitive performance when performed while walking (Suk et al., 2020). Using this cognitive task in an upper limb flexion-extension motor activity shows a high sensitivity to differentiate between the different states of cognitive impairment with the number of repetitions (speed) and the variability of the push-ups (Toosizadeh et al., 2019). This cognitive task shows significant results in 4 reviewed studies (Gillain et al., 2016; Montero-Odasso et al., 2017; De Cock et al., 2019; Åhman et al., 2020a). Also, cross-sectional studies have previously been studied using the variable “words per unit of time” (TUG + count back and TUG + name animals). It seems to be an excellent discriminator between the different levels of cognition: healthy, MCI, and dementia (Åhman et al., 2020b) and a possible good cognitive impairment predictor (Åhman et al., 2020a).

On the other hand, although the use of motion analysis platforms and body sensors allow an instrumentalized analysis of gait and a large number of parameters (Beauchet et al., 2017; Montero-Odasso et al., 2017; Ceïde et al., 2018), less expensive and infrastructure-free parameters are preferred for clinical use. One example is accelerometers, a device included in smartphones and smart bands (Gillain et al., 2016; Kikkert et al., 2017; Muurling et al., 2020). A recent study sheds some light on the gait parameters and variables that can be used to differentiate between the different stages of cognitive impairment, finding specific variables to differentiate between CS and MCI, CS and dementia, and MCI and dementia that could also be useful in the prediction of cognitive impairment: stride time, swing time, time of double support, stance time, step length, and stride time (Ghoraani et al., 2021).

This review focused on gait as a motor task in the DT paradigm. A proposed advantage of using a DT model is bringing the cognitive and motor systems close to the limit, eliminating the effect of gait variation during the previously observed day (Bessot et al., 2020). Even so, future research should consider using the upper extremities, as there is evidence to suggest that it may be a proper method for detecting cognitive impairment in a DT paradigm (Ehsani et al., 2019). Likewise, cognitive impairment and motor impairment trajectories could provide relevant and more significant information regarding its correlation with the progression to dementia (Montero-odasso et al., 2018). Besides, the age and cognitive level of subjects are factors to consider when planning a new study. Some studies suggest that the DT paradigm could have greater predictive and discriminatory power in younger people due to fewer comorbidities that can interfere with motor performance (Kikkert et al., 2017) and less cognitive impairment reflecting the ability to execute the double task (Nielsen et al., 2018).

### Strengths

This is the first systematic review that considers exclusively prospective design studies to evaluate the predictive capacity of cognitive impairment of DT in pre-dementia stages. It contributes to collecting, synthesizing, classifying, and summarizing the main studies oriented to DT gait as a predictive marker of cognitive impairment. This study's findings are replicable and relevant for the early clinical screening of cognitive impairment and can support future clinical and biomedical studies.

### Limitations and Projections

Because of the heterogeneity of the results, it is challenging to develop a meta-analysis using this data. This review could guide future studies to compare the use of DT more specifically as a predictive marker of cognitive impairment. Furthermore, it could consider more databases and software for database registration, such as Covidence. The keywords were oriented to the use of gait, but other motor tasks, cognitive tasks, and methodologies to study them could be included in new searches. For that purpose, it may be helpful to expand the research question beyond the utility of DT, determining if GS alone is enough for cognitive impairment screening or requires more complex instrumented gait variables. It would reduce the heterogeneity of the results and consider more comparable parameters. Considering the abovementioned, gait speed alone should not be sufficient. Also, it would be significant for clinical setting applications to determine the most promising cognitive performance task and the protocol with less variability and better reproducibility. Other studies could obtain the abovementioned considering the results of this review.

## Conclusion

The evidence collected in this review suggests that DT gait is a useful predictor of cognitive impairment better than single-task gait speed measurement alone. The DT paradigm is a simple and inexpensive evaluation that helps predict and discriminate between different degrees of cognitive impairment applicable in the clinic at the outpatient level. Future research could focus on the use of coefficient of variability (CoV), stride time, delta CoV stride time, variability, swing time, stride width, steps per meter, cycle time variability gait, stride width cost, step symmetry, speed cost, DT time, numbers of words during DT, words recited per 10 s during DT, counting backward DT time, and delta DT time as parameters to predict cognitive impairment. Likewise, they could compare the older and younger population with different levels of cognitive impairment since these characteristics seem to enhance the predictive power of DT. It is relevant to note that walking as a motor task is not the only model since other motor tasks have also shown promising results.

## Data Availability Statement

The original contributions presented in the study are included in the article/supplementary material, further inquiries can be directed to the corresponding author.

## Author Contributions

FR and MG conceived the idea, developed procedures for paper selection, data extraction, and drafted the manuscript. MG reviewed these procedures, developed the statistical analysis, and answered the reviewer's comments. FR coordinated, performed, reviewed paper selection, and data extraction. All authors critically reviewed, made important intellectual contributions to this manuscript, and read and approved the final manuscript.

## Funding

FR was funded by the Unidad de Postgrado, Facultad de Ciencias, Universidad Mayor APC grant for MSc studies research publication. MG was funded by Beca Especial de Apoyo DCBM tuition fee/stipend grant for Doctoral Studies 2021 from Escuela de Postgrado, Facultad de Medicina, Universidad de Chile.

## Conflict of Interest

The authors declare that the research was conducted in the absence of any commercial or financial relationships that could be construed as a potential conflict of interest.

## Publisher's Note

All claims expressed in this article are solely those of the authors and do not necessarily represent those of their affiliated organizations, or those of the publisher, the editors and the reviewers. Any product that may be evaluated in this article, or claim that may be made by its manufacturer, is not guaranteed or endorsed by the publisher.
